# Moderating effect of safety culture on the association inter work schedule and driving performance using the theory of situation awareness

**DOI:** 10.1016/j.heliyon.2022.e11289

**Published:** 2022-10-28

**Authors:** Al-Baraa Abdulrahman Al-Mekhlafi, Ahmad Shahrul Nizam Isha, Mohammed Abdulrab, Muhammad Ajmal, Noreen Kanwal

**Affiliations:** a Department of Management & Humanities, Universiti Teknologi PETRONAS, Seri Iskandar 32610 Perak, Malaysia; b Management Department, Community College of Qatar, Doha 00974, Qatar

**Keywords:** Safety culture, Driving performance, Work schedule, Oil and gas tanker driver

## Abstract

The adverse effects of work schedule on driving performance are relatively common. Therefore, it is necessary to fully understand an organisation’s safety culture to improve driver performance in order to avoid road crashes. This study aims to investigate the moderating role of safety culture in the relationship between driver work schedules and driving performance. The study developed a conceptual framework based on the literature review of existing studies, which is supported by situation awareness theory that explains the model’s relationships and supports the study’s hypotheses. Three hundred four questionnaires were collected from oil and gas truck drivers then Structural equation modelling (SEM) was applied to test the study hypotheses. Derived from the findings, the outer loading for all items was above the threshold of 0.70 unless two items were deleted. The latent exogenous variables of safety culture and work schedule explained 59.1% of driving performance. Besides, work schedule and safety culture significantly impact driving performance. In addition, the results show that safety culture moderates the unfavourable work schedule impact on driving performance with an effect size of 23%. Therefore, this study showed strong evidence that safety culture acts as a critical moderator in reducing the negative impact of work schedule on driving performance in the energy transportation sector. Drivers with high safety culture can manage and reduce the effect of work schedule disorder on driving performance through their safety attitude and patterns compared to those drivers with low safety culture. Consequently, the improvement in driving performance will be noticed among drivers with a high awareness of safety culture.

## 1 Introduction

Road transport firms are made up of multiple drivers who work outside of the workplace’s physical boundaries, which restricts the scope of supervisory control [1, 2]. Drivers usually take responsibility for their duties and can decide to face any road danger [3]. Consequently, the drivers gain skills and abilities that have the potential to influence the organisation’s overall safety. Therefore, all workers must be linked with the rest of the organisation in order to build a safety culture through all positions in the firm [4]. According to Reason and Hobbs [5], enhancing a safety culture requires all organisations' people to be involved in the procedure and committed to improving safety. Thus, the management should prioritise safety to build a high-safety culture. According to Mooren, Grzebieta [6] commitment of the management toward safety can reduce the impact of all levels of risks. Besides, an inadequate level of management’s commitment to safety will decrease the employee’s commitment to safety measures at work, which in turn will increase the possibility of work accidents [7]. At the same time, research by Nævestad, Hesjevoll [8] highlights that management commitment toward safety could help as a leading indicator for road safety. Nævestad, Blom [9] believed that a high safety culture within an organization could be considered as one of the critical factors that will reduce such accidents and improve overall road safety.

Therefore, to build a solid safety culture, an organisation must consider certain characteristics such as management commitment, good communication and improved employee safety patterns [10]. In particular, Reason and Hobbs [5] underline that employees must be aware of the dangers in their work and anticipate equipment and people’s mistakes in order to produce the proper action to avoid this danger. However, such awareness may be difficult to achieve when most of an organisation’s workers are outside the workplace [5]. Workplace safety could be at risk from several factors, including human, technological, organizational, and environmental factors. Based on situation awareness theory, managers and personnel in road transport firms must be aware of risk issues within the workplace, on the road, at loading/unloading locations, and outside of work time [11]. The necessity to disclose errors and near-misses is referred to as safety culture. Indeed, because most road transportation enterprises' tasks are performed outside the company, people in organizations must trust one another to reveal errors and near-misses to control potential risks. The organization may adjust work schedules and perhaps avoid areas and times where and when hazards arise if they understand when and where risk occurs [5].

Typically, working in road transportation is performed by shifts system. In many companies, an irregular work schedule poses a safety risk because it leads to low driving performance. Long-haul truck drivers (oil and gas) are one example of a transportation profession where shift work is common [12, 13]. Previous studies suggest that individuals with non-standard work schedules may be unable to satisfy their sleep needs, resulting in impaired driving performance [14]. Based on a survey of a random sample of European truck drivers [15], noted that drivers suffer from low performance based on their sleep needs because of their work schedule. Therefore, irregular shifts are rarely avoidable among those who work in the road transportation field. However, the negative impact of an irregular work schedule can be mitigated by employing effective strategies such as scheduling work and rest times to allow adequate sleep recovery, napping, and ingesting alertness-enhancing compounds such as caffeine. Thus, all these strategies cannot be successful if drivers' safety culture is low [16, 17]. Heavy truck drivers are one of the most significant drivers exposed to occupational injury because of road accidents [6, 18]. Heavy trucks in many countries are involved in severe and fatal road crashes [6]. In addition, truck drivers work in an environment that leads to poor performance since they might be away from home for days, work long hours, have irregular work schedules, and are under time pressure to deliver goods on time [19]; drivers are forced to wait in order to get admission to a loading terminal and usually stuck in traffic of the road [20, 21]; and some of the companies used to pay by miles driven rather than hours worked [22]. Furthermore, some drivers are required to load and unload freight alone [19, 23, 24, 25].

Because of the work schedule, circadian rhythm is important in the domain of driver fatigue. Truck drivers frequently work when they should be sleeping and sleep when they should be awake [26]. According to Mohamed, Mohd-Yusoff [27], most fatal incidents in Malaysia occur early morning hours and result in large injuries. Fatigue and stress are blamed for some of these accidents [26, 28]. Although several studies have examined the connection between a driver’s work schedule and their performance behind the wheel in different sectors [29, 30, 31, 32], there is a paucity of research on how safety culture can mitigate this unfavourable relationship between the work schedule and driving performance. Otherwise, previous studies in the oil and gas transportation sector addressed many important factors, such as exhaustion-related psychological risk factors [33], psychological well-being, perceived stress [34, 35], and fatigue assessment by the psychomotor vigilance test [36]. However, the safety culture’s role in regulating the work schedule balance and driving performance has been overlooked. Consequently, Malaysia’s oil and gas truck drivers must be addressed for their poor driving performance due to irregular work schedules and how a high level of safety culture may minimise this negative impact. This study intends to compensate for the lack of empirical evidence on the role of safety culture in moderating the association between work schedule and driving performance. Understanding the significance of improving driver safety culture will enable drivers to balance their work schedule and their abilities in order to avoid underperformance. Driver performance evaluation enhances the quality of service and reduces the likelihood of accidents occurring.

### 1.1 Research motivation and objectives

Heavy vehicle accidents are very dangerous. Its effects are very significant, especially the accidents of oil and gas tankers. The consequences of these accidents affect many road users, society, the health sector, the country’s economy, and the environment. Globally, traffic accidents have been estimated to cause about 1.2 million fatalities, 20–50 million terminal injuries, and about $ 518 billion yearly loss to material damage. Road accidents are especially common in low- and middle-income nations [37]. There is a particular concern for safety in heavy vehicles' transportation of hazardous materials because of their potential for fires, explosions, groundwater contamination, and toxic effects on human health if hazardous materials are spilt inadvertently or due to road accidents [38].

Moreover, the psychological impact of road traffic accidents impacts both direct participants and their families. Several nations have discovered that one of the benefits of minimizing road traffic accidents is that it reduces the cost of social support [39]. Accordingly, this was a strong motivation for us to study this problem and to provide recommendations that will help reduce these accidents in the future. Therefore, this study seeks to achieve several objectives. First, examine the work schedule’s impact on the driving performance among oil and gas tanker drivers. Second, to determine the effect of safety culture on driving performance. Third, investigate the moderating role of the safety culture in the negative relationship between work schedule and driving performance.

## 2 Literature review

The current study employed safety culture as a moderator to dampen the relationship between study variables. Previously, there has been an increase in literature on safety culture, which has been considered a moderator between different variables. For example, Wamid and Youssef [40] examined the moderating impact of safety culture on the relationship between safety climate, safety commitment, and safety behaviour. The study involved 250 employees in the oil products distribution company. Therefore, the results indicated that safety culture significantly moderates the relationship between study variables.

Similarly, Ahmed and Saba Waqas [41] conducted a study in Pakistan investigating the effect of safety culture on the association between occupational injuries and turnover intention among 111 employees in safety-sensitive fields. The results indicated the effect of occupational injuries on turnover intention. In contrast, a safety culture would not reduce turnover in Pakistan because of the lack of a prevailing safety environment. The conclusion was that a safe culture would not exist in Pakistan because of cultural differences. The study underlines how important it is to promote and preserve workers' physical, mental, and social well-being in a workplace health and safety system. Trinh and Feng [42] surveyed 78 construction projects in Vietnam. In construction projects, the complexity of the project and safety performance were investigated to see if a resilient safety culture had a role in mitigating the relationship between them. According to the findings, the project complexity variable has a detrimental influence on the performance of safety. Project complexity may have less influence on safety performance if there is a high level of safety culture.

### 2.1 Safety culture as moderating

The current study considered safety culture as the moderating variable on the association between the work schedule and driving performance for following justifications. First, the above-reviewed studies provided evidence that safety culture has been utilized as a moderator between several variables in different fields, such as: investigating the culture in the relationships between overall and life facet satisfaction among college students [43]. Investigation on how safety culture influences the connection between safety climate, safety commitment and safe behaviour among oil product distributors' workers [40]. The effect of safety culture in mitigating the association between workplace accidents and turnover intention in safety-sensitive domains is being investigated [41]. Fourth, construction projects looked at how a strong safety culture affects the relationship between the complexity of projects and safety outcomes [42]. Therefore, it can be concluded that the literature on the organization’s safety strategies suggests that safety culture has the potential power to moderate the relationship between the organization’s safety strategies to avoid occupational injuries [40, 41, 42]. Nevertheless, the studies have not gone into great detail on how safety culture affects the association between the schedule of work and driving performance, which is especially relevant in energy transportation firms. In the present research, a safety culture will help to reduce the unfavourable relationship between variables of this study.

The second point is that, according to Situation awareness theory [44], crashes can be avoided by going via 3 phases of situation awareness: identifying the aspects of the situation, (ii) understanding the present situation, and (iii) predicting future states in order to take appropriate action. In driving with a strong safety culture, the adverse influences of the work schedule on driving performance were more likely to be eliminated or reduced.

**Figure 1:**
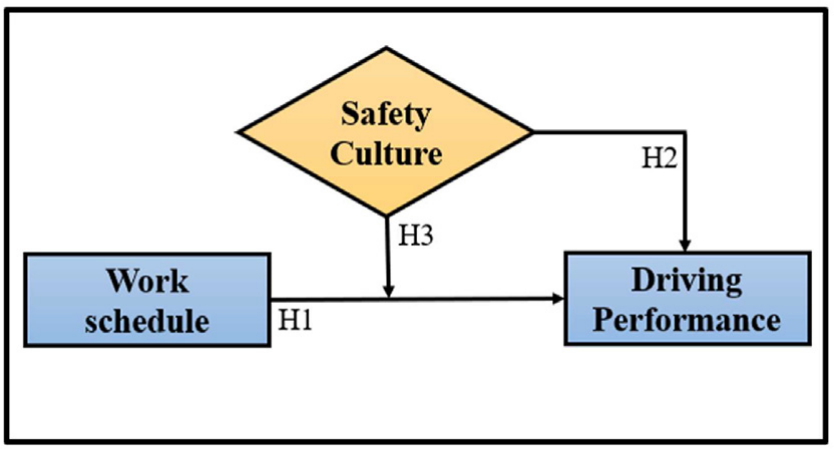
Conceptual framework.

Accordingly, Baron and Kenny [45] recommended that the weak or inconsistent results could be revitalized by introducing a moderating variable in a relationship between the two latent variables. When it comes to the connection between a driver’s performance and their work schedule, drivers with high safety culture can manage and reduce the effect of work schedule disorder on driving performance through their safety attitude and patterns compared to those drivers with low safety culture. Consequently, this study developed the conceptual framework, as seen in Figure 1, and proposed the following research hypotheses:

H1 The schedule of work has a significant influence on driving performance.

H2 Driving performance is significantly affected by a safety culture.

H3 The negative correlation between the schedule of work and driving performance diminishes as the level of safety culture increases among drivers.

### 2.2 Background theory (situation awareness theory)

According to Gilson [46], Oswald Boelke established the notion of situational awareness during World War I, he recognized the necessity of understanding the adversary before the enemy got similar knowledge and created ways to attain this. The separation between the human operator’s perception of the system state and natural system status is central to the definition of situational awareness [47]. The first impetus for R&D came from the aviation sector, where pilots and air traffic controllers are under intense pressure to improve their situational awareness [48]. Control measures based on incorrect situational awareness may exacerbate a poor occurrence. Such circumstances led up to the Chornobyl disaster [48].

The conceptual framework of this study has been developed based on situation awareness theory. According to Endsley (1995), understanding the situation elements concerning a proper execution on time is essential for risk and safety assessment. The environment where employees participate should be recognizable to anticipate what will happen next and produce appropriate action. This theory suggests that accidents can be prevented through three stages of situation awareness: (i) identifying the elements of the situation, (ii) understanding the current circumstance, and (iii) predicting future states to produce actions [44]. Based on this theory, safety culture characterizes drivers' skills and behaviour patterns in responding to road safety risks during their duty or outside the workplace. Hence, drivers with a high safety culture can overcome the negative influences of the work schedule on their performance. As a result, they are more likely to be eliminated or reduced. Thus, this theory reflects how driving performance is adversely affected by the driver’s work schedule and ability to deal with safety risks during driving duty, before and after the task. Therefore, the improvement in driving performance will be noticed among drivers with high abilities to manage safety risks as measured via the safety culture level. So, if the driver has a high level of safety culture, he will try to avoid anything that could impact his performance. For example, suppose the driver realizes that he has a trip early the following day (identify and understand the situation). In that case, he will go to bed early because going late will impact his performance the next day. Therefore, there is a high possibility of road accidents.

## 3 Research method

The present study is based on a quantitative research design since quantitative data were collected on the study variables utilising a cross-sectional design for the questionnaire study. As the study aims to verify the proposed relationships, a quantitative approach was deemed the most appropriate research method. Since this method is designed to test hypotheses, statistical tests and analyses are quantitative to confirm or disconfirm the hypotheses developed. A quantitative approach enables statistical analysis to ensure that the data collected are reliable and valid [49]. Furthermore, the quantitative design of the study allows the researcher to figure out that the whole population is representative at lower costs than data collection for the whole population [50].

### 3.1 Survey development

A survey questionnaire was developed based on the literature analysis to study the moderating influence of safety culture on the relationship that exists between oil tanker drivers' work schedules and their driving performance in Malaysia. The current study used a five-point Likert scale ranging from 1 = (Never) to 5 = (Always) with 41 items on the questionnaire [51, 52, 53, 54, 55, 56]. The Likert scale is widely used since it is one of the most trustworthy methods of measuring views, perceptions, and behaviours [57]. Table 1 depicts the organization of the research variables. Items of the research questionnaire are presented in the supplementary file.

**Table 1:** Organization instrument of the study.

Construct	Dimension	No. of Items	Reference
Work_schedule	Dayshift Nightshift Non-stander shift	15	[51, 52]
Driving _performance	Driver attention Driver vigilance Driver reaction time	11	[55, 56]
Safety_culture	Safety responsibility, communication, safety value and patterns, management attitudes	15	[54, 58]

### 3.2 Data collection and sampling

Three hundred fifty-seven surveys were delivered to drivers who work to transport energy from most of Malaysia’s regions using a random selection approach. The questionnaire was collected personally by visiting oil and gas company branches in the period from 2019 to 2020. Afterwards, invalid questionnaires were eliminated, and 304 valid questionnaires were gathered, yielding an 85.9 per cent response rate. According to Maccallum and Bryant [59], this sample size fits the SEM sample size criteria; the minimal sample size was determined utilizing Gpower software. The sample size was calculated with and power of 1- = 0.80. Therefore, the results indicated (N = 279) as a minimum sample, which is less than this study’s sample size (N = 304).

In the descriptive characteristics of the study sample, most drivers were Male, 99.7% because females avoid working in difficult work such as driving heavy vehicles. Regarding age, the highest percentage was in the group of drivers between 30 and 39 years, with 48.2%. At the same time, the highest rate of driver’s ethnicity was from Malay, with 93.8%. Finally, regarding the education level, most drivers hold Secondary education with 83.7%.

### 3.3 Ethics

Ethical approval for this study was obtained through the Department of Management & Humanities, Universiti Teknologi PETRONAS. As instructed by the department at the beginning of the survey, a brief introduction was added to let participants know the study’s objective and ask them to participate in this survey as volunteers. Correspondingly, we got informed consent from all subjects and guaranteed them confidentiality and anonymity.

### 3.4 Structural equation ModelingSEM

SEM is a multivariate analysis utilized to assess the validity of hypotheses by gathering samples pertaining to a theory or idea and then testing it [60, 61]. The two main approaches for SEM are partial least squares structural equation modelling (PLS-SEM) and covariance-based structural equation modelling (CB-SEM) [62, 63]. In describing the relationships between the study’s indicators and constructs, PLS-SEM seems more adaptable than CB-SEM [64]. PLS-SEM operates well in either size of the sample; however, this must meet the sample size’s minimum condition, allowing variables with intricate implications on particular model components to be generated [65]. The SEM method provides the following benefits: (i) SEM would be used to precisely evaluate complex model hypotheses based on many observations [66]. (ii) SEM perform well, particularly for complicated models with several indicators and latent variables [66]. Thus, SEM is now widely employed in various fields of social science investigation, such as hospitality management [67], the construction industry [68, 69], the petroleum industry [70], commercial aviation [71], education [72], safety and health [73, 74].

Consequently, the PLS-SEM approach was utilized in this work to test the three offered hypotheses. In addition, the Smart-PLS v3.2.1 software examined the path analysis and measurement model’s fitting.

## 4 Result

### 4.1 Measurement model

**Figure 2:**
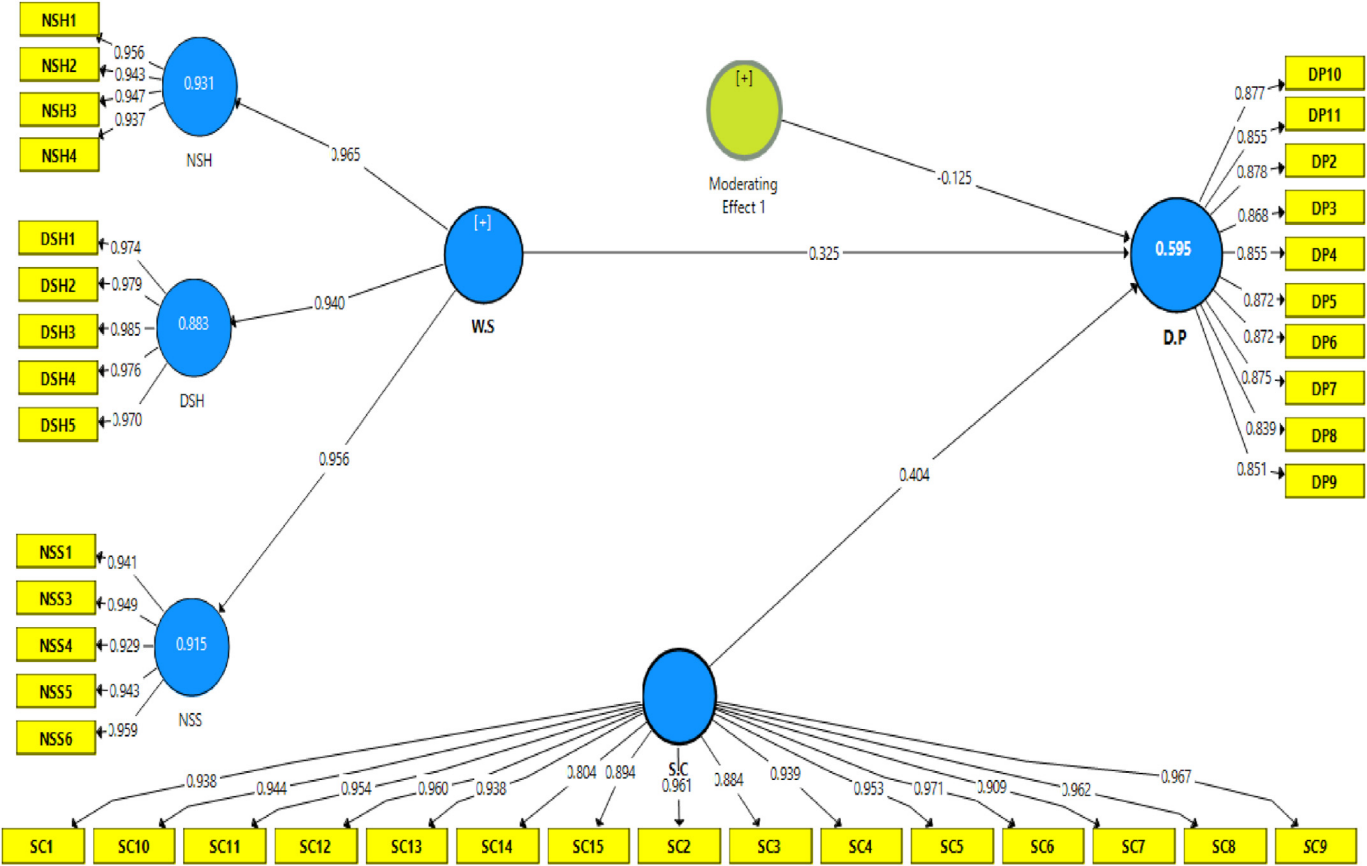
Measurement model.

Hair Jr, Hult [64], evaluating the measurement model entails estimating indicator reliability such as composite reliability, average variance extracted, and discriminant validity. In general, outer load signals between 0.40 and 0.70 must be deleted if removing the item significantly increases AVE and composite dependability [75]. The outer loadings values for all items in the measurement model were more than 0.70, as shown in Table 2 and Figure 2. As a result, more than 0.70 are appropriate for further investigation [64]. The internal consistency was tested by composite reliability (CR) for items outer loading. CR should be greater than 0.70 [64]. As shown in Table 2, all items in the model met the CR > 0.70 benchmark and were thus acceptable. With values over 0.50, AVE is a typical metric for measuring convergent validity in model constructs; this suggests that, as Wong [76] indicated that 0.50 is acceptable convergent validity. The results in Table 2 show that these tests were passed for the measurement model of the study constructs.

**Table 2:** Convergent validity.

Construct	Items	Outer load	Cronbach’s Alpha	Composite reliability	AVE
Safety-culture	SC_1	0.938	0.989	0.990	0.870
	SC_2	0.961			
	SC_3	0.884			
	SC_4	0.939			
	SC_5	0.953			
	SC_6	0.971			
	SC_7	0.909			
	SC_8	0.962			
	SC_9	0.967			
	SC_10	0.944			
	SC_11	0.954			
	SC_12	0.960			
	SC_13	0.938			
	SC_14	0.804			
	SC_15	0.894			
Driving-performance	DP_1	deleted	0.962	0.967	0.747
	DP_2	0.878			
	DP_3	0.868			
	DP_4	0.855			
	DP_5	0.872			
	DP_6	0.872			
	DP_7	0.875			
	DP_8	0.839			
	DP_9	0.851			
	DP_10	0.877			
	DP_11	0.855			
Work-schedule	NSH-1	0.956	0.975	0.979	0.891
	NSH-2	0.943			
	NSH-3	0.947			
	NSH-4	0.937			
	DSH-1	0.974			
	DSH-2	0.979			
	DSH-3	0.985			
	DSH-4	0.976			
	DSH-5	0.970			
	NSS-1	0.941			
	NSS-2	Deleted			
	NSS-3	0.949			
	NSS-4	0.929			
	NSS-5	0.943			
	NSS-6	0.959

According to the observed standards, discrimination validity is a concept that differs from the other constructions [77]. It used Fornell Larcker’s (1981) and Cross Loading criteria to assess discriminant validity. According to Fornell and Larcker [78], the AVE’s square root should be larger than the latent variable correlation. Table 3 shows the findings of the discriminative validity of the measurement model [79].

**Table 3:** Discriminant validity (Fornell-Larcker).

Constructs	D.P	DSH	NSH	NSS	S.C
D.P	0.864				
DSH	0.641	0.977			
NSH	0.681	0.864	0.946		
NSS	0.670	0.843	0.895	0.944	
S.C	0.744	0.735	0.818	0.778	0.933

The second approach utilised in this research is the cross-loading criteria, which was also utilized to verify discrimination validity. This method defines that the indicators loading on a particular construct should be larger than the loading of all other constructs per line. In addition, the loading of the main build of signs or objects must be greater than the loading of different constructions. The results in Table 4 reveal that (indicators) latent variables have a higher loading than the other variables by row. Furthermore, the results for each construct demonstrated a significant degree of one-dimensionality.

**Table 4:** Discriminant validity by cross-loadings.

Items	D.P	DSH	NSH	NSS	S.C
DP10	**0.877**	0.552	0.578	0.587	0.641
DP11	**0.855**	0.513	0.557	0.546	0.607
DP2	**0.878**	0.590	0.617	0.595	0.660
DP3	**0.868**	0.543	0.586	0.574	0.650
DP4	**0.855**	0.553	0.597	0.571	0.652
DP5	**0.872**	0.577	0.607	0.586	0.659
DP6	**0.872**	0.557	0.589	0.584	0.656
DP7	**0.875**	0.555	0.598	0.577	0.660
DP8	**0.839**	0.533	0.570	0.589	0.628
DP9	**0.851**	0.570	0.583	0.580	0.611
DSH1	0.617	**0.974**	0.844	0.810	0.711
DSH2	0.620	**0.979**	0.846	0.830	0.717
DSH3	0.632	**0.985**	0.846	0.834	0.718
DSH4	0.630	**0.976**	0.84	0.809	0.724
DSH5	0.634	**0.970**	0.843	0.834	0.720
NSH1	0.642	0.845	**0.956**	0.856	0.777
NSH2	0.644	0.796	**0.943**	0.851	0.773
NSH3	0.645	0.846	**0.947**	0.848	0.763
NSH4	0.644	0.778	**0.937**	0.831	0.783
NSS1	0.652	0.804	0.855	**0.941**	0.741
NSS3	0.592	0.810	0.853	**0.949**	0.737
NSS4	0.673	0.798	0.842	**0.929**	0.729
NSS5	0.629	0.771	0.837	**0.943**	0.729
NSS6	0.616	0.796	0.838	**0.959**	0.739
SC1	0.665	0.691	0.779	0.723	**0.938**
SC10	0.72	0.695	0.751	0.744	**0.944**
SC11	0.704	0.682	0.768	0.731	**0.954**
SC12	0.664	0.703	0.779	0.739	**0.960**
SC13	0.736	0.686	0.748	0.739	**0.938**
SC14	0.725	0.600	0.679	0.634	**0.804**
SC15	0.757	0.622	0.724	0.698	**0.894**
SC2	0.682	0.693	0.785	0.736	**0.961**
SC3	0.697	0.699	0.759	0.715	**0.884**
SC4	0.684	0.694	0.772	0.75	**0.939**
SC5	0.65	0.703	0.765	0.735	**0.953**
SC6	0.655	0.693	0.785	0.733	**0.971**
SC7	0.709	0.726	0.787	0.74	**0.909**
SC8	0.637	0.681	0.770	0.723	**0.962**
SC9	0.672	0.698	0.785	0.732	**0.967**

### 4.2 Structural model

#### 4.2.1 Direct relationships

Path analyses are a type of linear regression statistical approach. Path analysis is favoured for social science and analytical management methodologies. Likewise, path coefficient analysis is a dominant tool for examining complex relations simultaneously [80]. Following the fitting of the model, structural equation modelling may be used to investigate the connections between study variables. The structural model describes the connections between research variables [81]. The findings show a link between exogenous factors and endogenous variables. The fundamental focus of the structural model evaluation is the fit of the whole model, including posited parameter values, dimensions, path, and significance [81].

**Figure 3:**
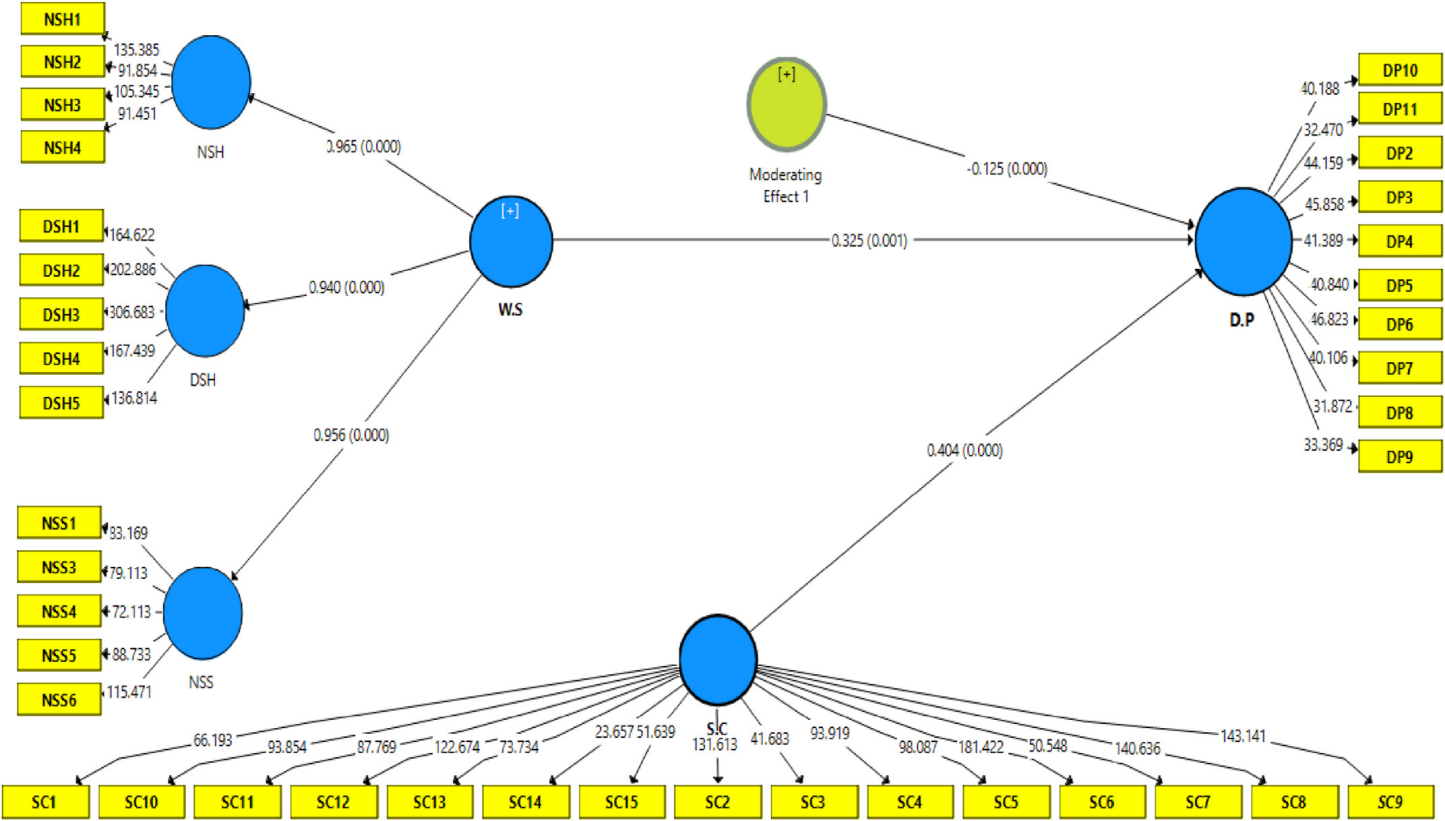
Structure model.

Following the study context in this model, PLS-SEM was used to evaluate the moderating influence of safety culture on the link between work schedule and driving performance. The bootstrapping method was used to determine the significance of the model hypothesis. Bootstrapping is used in the random evaluation of the original data to create samples equivalent to the original data. This approach evaluates the data’s dependability and predictive power, as well as the inaccuracy of the measured path coefficient [82]. The dependent construct was examined for standardised path coefficients (***β***) and p-values, as shown in Figure 3, Table 5. The results revealed that work schedule substantially influenced driving performance (***β*** = 0.325, p < 0.001).

Similarly, safety culture substantially impacts driving performance (***β*** = 0.404, p < 0.000). Furthermore, effect size has been utilised to assess the significance of each independent variable’s impact on a dependent variable [83]. Effect size values F^2^: 0.02, 0.15, and 0.35 are measured as minor, moderate, and strong, respectively, in the context of a statistical study [77]. Table 5 shows that the impact sizes between factors were small.

**Table 5:** Hypothesis and path coefficient.

Hypotheses	H1	H2
**Path Relationships**	S.C→ D.P	W.S→ D.P
**Path coefficient (β)**	0.404	0.325
**Standard Error**	0.092	0.093
**F2 Value Effect**	0.107	0.085
	Small	Small
**t Values**	4.404	3.483
**p Value**	0.000	0.001
**Level of Significance**	∗∗∗	∗∗∗
**Results**	Supported	Supported

#### 4.2.2 Explanatory power

The data show that the measuring model has good reliability of items, convergent validity, and discriminant validity. In addition, the PLS technique yielded multiple squared (R^2^) correlations for the model’s endogenous variables. ***R***^***2***^ is treated in the SEM-PLS method in the same way as in traditional regression [82].

The ***R***^***2***^ is defined as how independent variables all together interpret the variance of the dependent variable. Therefore, a greater R^2^ value increases the structural model’s ability to forecast. The R^2^ values were obtained using the PLS technique in this investigation, as indicated in Table 6. In his model, the R^2^ value for the dependent variable (driving performance) was 0.591, suggesting that the latent exogenous variables of safety culture and work schedule could explain 59.1% of driving performance. Based on the Chin [82] guidelines, the R^2^ value of 59.1% is substantial.

**Table 6:** Explanatory power *R*^2^.

Endogenous Variable	*R*^*2*^	Adj *R*^*2*^	Explained
Driving performance	0.595	0.591	Substantial

#### 4.2.3 Moderating effect analysis

The moderator is defined as the variable that influences the strength or weakness of the relationships/interaction between independent variables or a predictor and a dependent variable or a criterion. In particular, the moderator is a third variable within a relationship research framework that influences the zero-order correlation between two other variables [45]. PLS-SEM was utilized in the current study to define and estimate the interplay of the moderating role of safety culture on the link between work schedule and poor driving performance [84, 85]. The product indicator technique was utilized in this study since the postulated moderating variable (safety culture) is continuous in nature [86]. According to Henseler and Fassott [85], considering that the outcomes of the product indicator technique are generally equivalent or superior to those of the group comparison strategy, we propose utilising the product indicator approach at all times.

**Figure 4:**
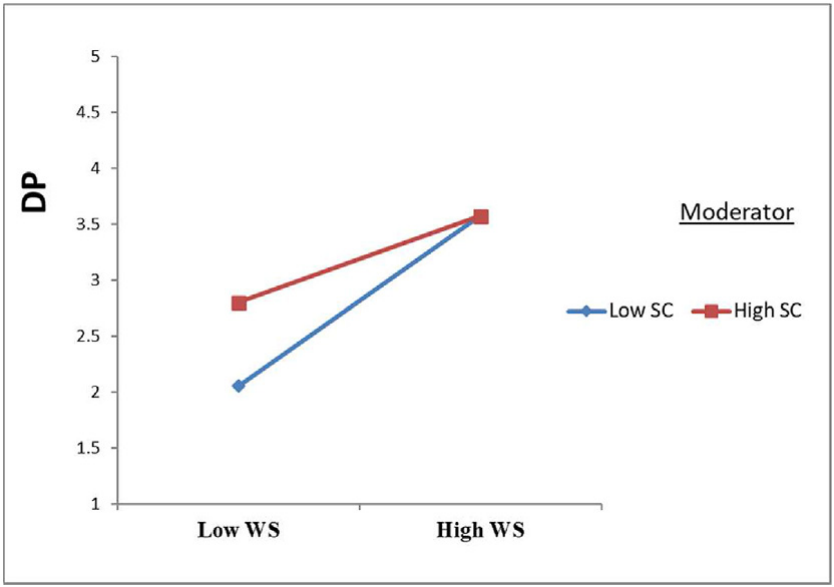
Interaction Effect of Safety Culture (S.C) between Work schedule (W.S) and Driving Performance (D.P).

As previously mentioned in hypothesis 3, the safety culture moderates the association between schedule work and drivers' performance. Table 7 and Figure 4 show that the interaction factors representing the work schedule x safety culture x driving performance were significant (β = −0.125, t = 3.548, p < 0.000), as expected. As a result, hypothesis 3 was completely validated, following the principles of Aiken, West [87] and Marcus, Schuler [88]. The path coefficient information was used to illustrate the moderating influence of safety culture on the relationship between study variables. Figure 4 states that incorporating the moderate effect of safety culture may mitigate the unfavourable association between work schedule and driving performance.

**Table 7:** Moderating effect.

Hypothesis	Relationship	B	S.E	T-Val.	P-Va.	Decision
H3	W.S→S.C→D.P	-0.125	0.035	3.548	0.000	Supported

### 4.3 Identifying the strength of moderating effects

The current study calculates the effect sizes using Cohen (1988) guidelines to evaluate the strength of safety culture as a moderator that affects the association between the schedule of drivers and their driving performance. Furthermore, the determined strength of moderating impact could be assessed by comparing the R2 value of the main model (without moderator) to the R2 value of the entire model, which included exogenous and moderator effects [85]. Thus, this study determines the strength of moderation effects via the underlined formula [85, 89].

0.02, 0.15, and 0.35 are classified as minor, moderate, and large moderating effect sizes [89]. It is worth noting that Chin, Marcolin [90] remarked that a moderate effect size somehow doesn’t necessarily suggest that the moderate impact is significant. If the ensuing beta changes are considerable, even a little interaction impact might be important; these conditions must be addressed [90]. Based on the guidelines of [85, 89], the strength of the moderation impact of safety culture was determined. Table 8 implied the driving performance effect size was 0.232, which reflects that the effect size of moderation impact is moderate [91].

**Table 8:** Effect size of the moderating impact.

Dependent Variable	R^2^		F^2^	Explained Effect-Size
	Included	Excluded		
Driving Performance	0.591	0.496	0.232	Moderate

## 5 Discussion

Based on the findings, the current study discovered that work schedule affected driving performance as objective one (H1). This conclusion is consistent with earlier researchers [16, 51, 92, 93, 94, 95], suggesting that work schedule has a major impact on employee performance. Based on the results, drivers who understand the influence of work schedules on their performance while driving would better prevent work schedule disorder. Given that drivers spend the majority of their time on the road, one may claim that there is a chance that they will be impaired in mental or physical functioning through their driving shift. This raises the probability of traffic crashes, either because of their shift period or because they work a non-standard shift [96]. Similarly, this study found that shifts impacted most drivers because of the lengthy haulage of oil and gas driving tasks. As a result, this will directly influence driver alertness, resulting in poor performance. Besides, keeping attention necessitates regular self-regulation on the part of the driver. Therefore, the driver must balance the subjective costs (effort exertion) and advantages (intrinsic and extrinsic incentives) of keeping careful attention over time [97].

This study discovered a significant association between safety culture and driving performance as objective two (H2). The findings are consistent with previous research [98, 99, 100]. As a result, the safety of a driving journey is mostly dependent on the driver’s performance. The driver is the primary human operating a vehicle, and their responsibilities are pretty demanding since they must meet the numerous demands and requests linked with their driving job [101]. They must also maintain their driving abilities, particularly for trains and commercial vehicles, as well as be vigilant and ecologically conscious when driving [102].

The major purpose of the present research is to evaluate the moderating role of safety culture on the negative impact of work schedules on driving performance. According to objective three (H3), safety culture moderates the association between work schedule and driving performance. PLS path modelling findings validated this hypothesis. This research supports the awareness situation theory, arguing that there must be a ‘fit’ between knowing the current situation and forecasting future requirements to produce activities that maintain attention for a lengthy period of time when driving [44]. According to situation awareness theory, the moderating influence of safety culture on the association between the driver’s schedule and the performance of drivers may be described using situation awareness theory [44]. suggested that awareness of scenario factors about the correct action at the right moment is critical for risk management and safety. Drivers must be able to perceive their surroundings in order to foresee what will happen next and, consequently, take the right action. This hypothesis explains how, as a moderator, safety culture minimizes the amount to which the work schedule negatively impacts driving performance. It was closely tied to its drivers' skills to control safety hazards while driving, before duty, and after work. Therefore, the improvement in driving performance will be noticed among drivers who are aware of the safety culture to manage hazards.

Finally, the magnitude of the moderating effect was assessed in this study by comparing the R2 value of the main model (without moderator) to the R2 value of the complete model, which included IV, DV, and moderator [85, 89]. Therefore, based on the results, the current study demonstrates that the effect size of safety culture intervention decreased the magnitude of the unfavorable relationship between work schedule and driving performance was 23.2%, which is considered a moderate effect size based on Cohen [89] guidelines. Therefore, the current study found substantial evidence that safety culture operates as a crucial moderator, which contributes to dampening the unfavorable association between the effect of schedule of drivers' work and the performance of drivers among oil and gas truck drivers.

Based on the results of the current study, many recommendations to enhance the safety culture among Malaysian oil and gas tanker drivers have been acknowledged. First and foremost, commitment and communication are essential components of a successful and good safety culture. A solid safety culture requires open and honest communication between drivers and supervisors [103]. Second, safety training assists oil and gas drivers in understanding safe behaviours and expectations [104, 105]. Third, involve drivers. Research indicates that firms with a strong safety culture keeping employees engaged and emotionally committed to the company and its goals [106].

## 6 Conclusion

As per the results and discussion mentioned above, this study aimed to study the moderating impact of safety culture in the association between work schedule and driving performance. The results have proved that work schedule and safety culture significantly influence driving performance. Besides, the finding proved the moderating impact of safety culture as a preventative intervention that mitigates the unfavorable association between work schedule and driving performance.

### 6.1 Research contributions

As a result, this study’s findings might have theoretical and practical ramifications. Regarding the theoretical viewpoint,

• The current study expands the situation awareness theory by establishing a comprehensive moderating model that combines diverse safety culture views.
• In addition, studying the moderating influence of safety culture in the association between study variables will contribute to expanding the current body of knowledge.

While in terms of practical consequences,

• This study is one of the first studies that gave practical evidence to energy transportation firms to assist them in making decisions regarding the redesign of the work schedule for drivers and promoting drivers' safety culture to prevent poor driving perform-ance.
• Besides, this research found evidence of the importance of each variable studied, which motivates immediate administrators of drivers to establish evaluation criteria and identify the importance of regulating the work schedule and enhancing safety culture, resulting in a reduction or increment in driving performance.

### 6.2 Study limitations and future research

Although the study provided significant contributions, the study’s limitations have been acknowledged. Because this study relied on data collected from oil and gas tanker drivers to evaluate their perspectives on study variables, it cannot be generalized to other heavy vehicle industries due to differences in work situations and cultures. In addition, even though the sample size met the requirement of Krejcie and Morgan [107], it is preferred to make it bigger in future studies. Besides, the study did not consider the change in the oil and gas market after the Covid-19 pandemic, the demand increasing, which in turn creates new conditions for drivers that need to be addressed. Finally, as observed during the data collection period, some intriguing topics for future research include psychological hazards, job load and sleep disorders among oil and gas truck drivers. Furthermore, it is valuable to investigate the impact of the work environment on the driver’s performance.

## Declarations

### Author contribution statement

Dr. Al-Baraa Abdulrahmam Al-Mekhlafi: Conceived and designed the experiments; Performed the experiments; Analyzed and interpreted the data; Contributed reagents, materials, analysis tools or data; Wrote the paper.

Dr Ahmad Shahrul Nizam Isha: Conceived and designed the experiments; Performed the experiments; Contributed reagents, materials, analysis tools or data; Wrote the paper.

Dr. Mohammed Abdulrab: Analyzed and interpreted the data; Wrote the paper.

Muhammad Ajmal; Noreen Kanwal: Contributed reagents, materials, analysis tools or data; Wrote the paper.

### Funding statement

Ahmad Shahrul Nizam Isha was supported by 10.13039/501100005710Universiti Teknologi Petronas [10.13039/501100016152YUTP- Cost centre 015LC0-358].

### Data availability statement

Data will be made available on request.

### Declaration of interest’s statement

The authors declare no conflict of interest.

### Additional information

No additional information is available for this paper.
