# Supplementary material for: Moderating effect of safety culture on the association inter work schedule and driving performance using the theory of situation awareness
Source: Heliyon. 2022 Oct 28;8(11):e11289. doi: 10.1016/j.heliyon.2022.e11289 (PMC9638756; doi:10.1016/j.heliyon.2022.e11289)
Supplement: supplementary material [file mmc1.docx]

**Questionnaire Items**

| **Construct** | **CODE** | **Item** |
| --- | --- | --- |
| Work Schedule | | |
| Night shift | NSH-1 | I have no experience sleepiness while driving. |
|  | NSH-2 | I have no experience decreased in my physical or mental functioning during the time I were awake. |
|  | NSH-3 | I have no experience sleepiness while commuting to my home after working the night shift. |
|  | NSH-4 | During night shift, I do not experience doze off while driving. |
| Day shift | DSH-1 | I don't have sleep problems such as snoring or obstructive sleep apnea |
|  | DSH-2 | During the day shift, I do not experience doze off while driving. |
|  | DSH-3 | During the day shift, I have no experience decreased in my physical or mental functioning during the time I were awake. |
|  | DSH-4 | During the day shift, I have no experience sleepiness while driving. |
|  | DSH-5 | I have no experience the time delay in getting to sleep at bedtime |
| Non-standard shifts | NNS-1 | The overall amount of sleep was somewhat insufficient. |
|  | NNS-2 | I have no experience sleepiness while driving. |
|  | NNS-3 | I do not have a problem falling asleep at bedtime |
|  | NNS-4 | I do not have a problem with waking up too early and not being able to get back to sleep |
|  | NNS-5 | During my break, I have no experience sleepiness during the time I were awake. |
|  | NNS-6 | During my break, I do not have a problem falling asleep at bedtime. |
| Safety culture | CS_1 | Concern about the possibility of killing or injuring persons |
|  | SC_2 | Concern about being assaulted (collision) |
|  | SC_3 | Concern about slipping when climbing in and out of the cabin |
|  | SC_4 | Does safety have a high priority within the company? |
|  | SC_5 | In my workplace, management ignores safety issues |
|  | SC_6 | My manager consults me to assist in resolving workplace problems |
|  | SC_7 | Management react quickly to any safety concerns |
|  | SC_8 | My manager always informs me about relevant safety issues |
|  | SC_9 | I am encouraged to offer ideas on safety |
|  | SC_10 | If I report a safety issue, I feel I am blamed for the problem |
|  | SC_11 | I can approach my manager to discuss problems regarding work |
|  | SC_12 | There are not always enough people to do the job safely |
|  | SC_13 | Feedback from any safety incident is good |
|  | SC_14 | I am unable to do my job if I follow procedures and rules exactly |
|  | SC_15 | Training covers the safety-critical aspects of the job |
| Driving performance | DP_1 | Operating entertainment systems do not distract me from driving (e.g., playing radio). |
|  | DP_2 | Operating navigation systems do not distract me from driving. |
|  | DP_3 | I sometimes push the wrong pedal |
|  | DP_4 | My reactions are faster than they used to be (e.g., braking in an emergency). |
|  | DP_5 | I sometimes cannot judge my speed. |
|  | DP_6 | I have no difficulty judging the speed of oncoming vehicles. |
|  | DP_7 | I have no trouble judging the distance from the vehicle in front. |
|  | DP_8 | I have no difficulty with identifying and reading road signs. |
|  | DP_9 | I sometimes cannot hear the horns of other vehicles/sirens from emergency vehicles |
|  | DP_10 | Sometimes my speedometer is hard to read during the daytime. |
|  | DP_11 | Sometimes my speedometer is hard to read during the night time |
